# Durable response to immunotherapy plus chemotherapy in a patient with untreated, brain-metastatic, *EGFR* exon 20 insertion mutation lung adenocarcinoma: A case report

**DOI:** 10.1097/MD.0000000000026650

**Published:** 2021-07-23

**Authors:** Jingying Nong, Yanfei Gu, Shuyang Yao, Yi Zhang

**Affiliations:** a Department of Thoracic Surgery, Xuanwu Hospital, Cancer Center of National Clinical Research Center for Geriatric Diseases, Diagnostic and Treatment Center of Lung Cancer, Capital Medical University, Beijing, China.; b Beijing United Family Healthcare Hospital, Beijing, China.

**Keywords:** *EGFR* rare mutations, immune checkpoint inhibitors, lung cancer

## Abstract

**Rational:**

Epidermal growth factor receptor (*EGFR*) 20 exon insertion is the second most common *EGFR* aberrations in non-small cell lung cancer (NSCLC). Despite some novel *EGFR* inhibitors, clinically obtainable management for this subset of patients remains an unmet need. there are no previous reports of upfront combination therapy with immunotherapy and chemotherapy for lung adenocarcinoma with brain metastasis harboring *EGFR* 20 insertion.

**Patient concerns:**

A 56-year-old man who sought care for dry cough was diagnosed with lung adenocarcinoma with brain metastases indicating a poor prognosis.

**Diagnosis:**

Next-generation sequencing of lung biopsied tissue revealed an *EGFR* exon 20 in-frame insertion (P772_H773insYNP+H773Y).

**Interventions:**

The patient started treatment of pemetrexed and carboplatin plus programmed cell death-1 inhibitor sintilimab in November 2019.

**Outcomes:**

The patient achieved partial responses both intra- and extra-cranially. After 6 cycles of treatment, the patient accepted sintilimab plus pemetrexed every 3 weeks as maintenance therapy, which was well-tolerated without any toxicity and is still ongoing after 18 months since initiation of 1st-line treatment.

**Lessons:**

This is the first case report of the clinical benefit of upfront immune checkpoint inhibitors (ICIs) plus chemotherapy for a brain metastatic NSCLC patient harboring *EGFR* exon 20 insertion mutation. Further study is needed to validate the predictor involved in responders to ICIs-based therapy with *EGFR* mutations.

## 1 Introduction

Programmed cell death-1 (PD-1)/programmed cell death-ligand 1 (PD-L1) immune checkpoint inhibitors (ICIs), alone or in combination with chemotherapy, have revolutionized the standard treatment for non-small-cell lung cancer (NSCLC), bringing unprecedented durable clinical benefit to late-stage patients. Despite this, a portion of patients displays only modest responses or remains unresponsive.^[1,2]^ Increasing evidence indicates epidermal growth factor receptor (*EGFR*) mutation to be a negative predictive factor for immunotherapy, with most data explored in classic *EGFR* mutations 19del and L858R,^[3,4]^ while data in patients harboring *EGFR* exon 20 insertion mutation are lacking. High-throughput sequencing such as next-generation sequencing could provide more information for treatment selection.

Here we describe a rare case of an advanced-stage lung adenocarcinoma patient with brain metastases (BM) harboring *EGFR* exon 20 insertion mutation who responded to 1st-line PD-1 inhibitor sintilimab plus chemotherapy (pemetrexed and carboplatin), with a continuous partial response both cranial and extra-cranial and progress free survival beyond 18 months.

## 2 Case presentation

**Figure 1:**
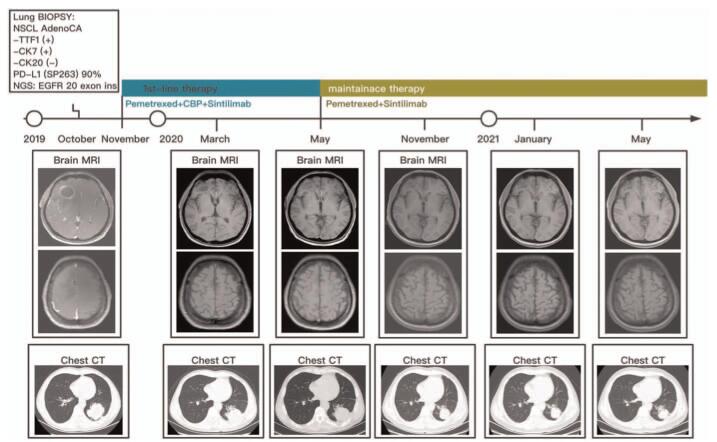
Timeline of clinical response. Shown is the timeline of cranial and extra-cranial clinical response of the NSCLC patient with EGFR 20 exon insertion mutant to 1st-line PD-1 inhibitor plus chemotherapy. The evolution of the disease was demonstrated by brain MRI and chest CT at various time points. NGS analyses were performed at the time of diagnosis (October 2019). Sintilimab plus chemotherapy (pemetrexed+carboplatin) were initially provided for 6 cycles, followed by Sintilimab plus pemetrexed as maintenance therapy.

**Figure 2:**
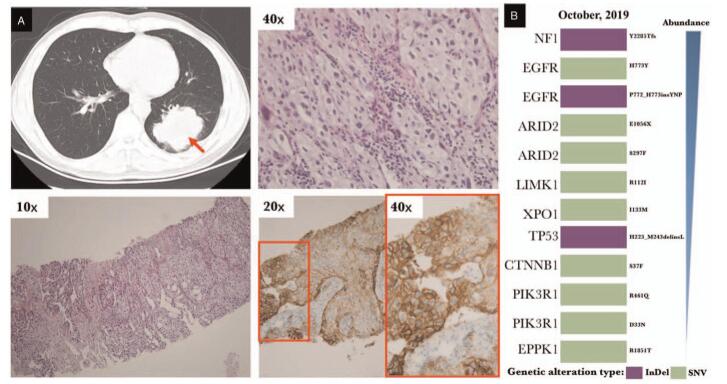
Histopathologic analyses and Molecular detection by NGS at diagnosis. (A) Histopathologic analyses at diagnosis. (upper left) CT imaging shows the biopsy site (arrowhead) at the left lower lobe mass of the lung. (lower left) Low-power magnification (original magnification, 10×) shows lung biopsy widely infiltrated by adenocarcinomatous architecture. (upper right) High-power magnification (original magnification, 40×) shows neoplastic cells with abundant pale eosinophilic cytoplasm and atypical round to oval nuclei. (lower right) 90% of these neoplastic cells express PD-L1. (B) Molecular alterations of lung biopsy by NGS at diagnosis.

This case was approved by the Ethics Committee, and written informed consent was obtained from the patient for the publication of this case report and accompanying images. Figure 1 shows the timeline of a 56-year-old man who sought care for dry cough that had been progressively worsening over 4 weeks, he was neurologically asymptomatic and had tobacco use of 1 pack × 20 years. A computed tomographic scan revealed a large mass in the left lower lobe of the lung. Magnetic resonance imaging (MRI) of the head revealed numerous tumor lesions, with mass effect and local edema. A core biopsy specimen of the left lower lobe mass showed lung adenocarcinoma that was positive for cytokeratin 7 (CK7), negative for cytokeratin 20 (CK20), and positive for thyroid transcription factor 1 on immunoperoxidase staining. The PD-L1 (SP263) testing showed high expression with a tumor proportion score of 90% (Fig. 2A). The patient was a stage IV lung adenocarcinoma according to TNM staging 8^th^ edition.

Molecular testing on the biopsied tissue via next-generation sequencing (NGS) revealed an *EGFR* exon 20 in-frame insertion (P772_H773insYNP+H773Y), Microsatellite stability, and high tumor mutational burden (TMB) 10.5 Mut/Mb. Also, alterations in immune-related genes were revealed including *CTNNB1* (Catenin β1) S37F, and *ARID2* (AT-rich interactive domain-containing protein 2) E1056X, with some variants of unknown significance (Fig. 2B).

The patient started treatment of chemotherapy (pemetrexed 500 mg/m^2^ and carboplatin AUC = 5) plus PD-1 inhibitor sintilimab 200 mg per 3 weeks in November 2019. After 4 cycles of treatment, the patient achieved a partial response per Response Evaluation Criteria In Solid Tumors, Version 1.1. He was in a good performance status without experience of any treatment-related adverse event except for grade II neutropenia. After 6 cycles of treatment, the patient then accepted sintilimab 200 mg plus pemetrexed 500 mg/m^2^ every 3 weeks as maintenance therapy, which was well-tolerated without any toxicity.

Follow-up imaging, by brain MRI, chest, abdominal, pelvic CT, and bone-scan at various time points showed a continuous decrease of both the lung and brain lesions, with no new sites of disease (Fig. 1). By the last follow-up in May 2021, the patient had achieved a PFS of 18 months. The treatment with pemetrexed plus PD-1 inhibitor is still ongoing since initiation of 1st-line treatment without evidence of progressive disease or any toxicity.

## 3 Discussion

*EGFR* mutations are detected in 20% to 50% of lung adenocarcinoma patients, *EGFR* exon 20 insertions are the next most common *EGFR* mutations in NSCLC after classical mutations, accounting for 4% to 10% of all observed *EGFR* aberration.^[5,6]^ Most *EGFR* exon 20 insertion mutations predict resistance to clinically achievable levels of tyrosine kinase inhibitors (TKIs) in advanced NSCLC, although there are rare exceptions. Some novel EGFR inhibitors have shown encouraging antitumor activity for *EGFR* exon 20 insertions,^[7]^ but commercially obtainable effective targeted drugs remain an unmet need in clinical management. Therefore, platinum-based chemotherapy is considered standard treatment for these patients.

This patient harbored *EGFR* exon 20 in-frame insertion (P772_H773insYNP+H773Y). exon 20 H773Y was previously detected combined with exon 20 insertions.^[6,8]^ Preclinical research indicated that the complex mutations of exon 20 insertion combined with H773Y resist 1st-generation EGFR-TKIs,^[8]^ while clinical evidence for the effect of 2nd or 3rd generation EGFR-TKIs are lacking.

Although ICIs have revolutionized the systemic treatment of patients of NSCLC with *EGFR*^wild-type (WT)^, the activity and clinical impact in the subgroup of *EGFR* mutation-positive tumors have been lower than in the *EGFR*^WT^ population in large 2nd- and 3rd-line phase III trials and 1st-line trials evaluating the efficacy of ICIs often excluded *EGFR-*mutation-positive patients. Anecdotal evidence suggests that some *EGFR* mutation-positive patients benefit from PD-1/PD-L1 inhibitors.^[9]^*EGFR* exon 20 mutations demonstrated a higher response rate and longer survival compared to classic *EGFR* mutations.^[10]^ A case report demonstrated that a heavily pre-treated patient of NSCLC with *EGFR* exon 20 insertion mutation responded to ICIs-based therapy.^[11]^ But the effect of upfront ICIs in lung cancer patients harboring *EGFR* exon 20 insertion mutation is lacking. This patient had high PD-L1 expression. PD-L1 is a positive predictive biomarker of ICIs, which has been prospectively validated and approved by the Food and Drug Administration, and was also positively associated with outcomes for ICIs in *EGFR* mutant NSCLC.^[4]^

Considering this patient was neurologically asymptomatic, with rare *EGFR* mutations, high PD-L1 expression, and High TMB,^[12]^ therefore, 1st-line immunotherapy plus chemotherapy was applied. A durable response was achieved with impressive control in both extra-cranial and cranial tumor lesions.

Brain metastases, associated with poor clinical outcomes, are diagnosed in approximately 20% of NSCLC patients.^[13]^ For asymptomatic BM, upfront systemic chemotherapy could be an option. However, traditional chemotherapy drugs have a limited role in BM management, owing to the presence of efflux pumps,^[14,15]^ and lacking penetration of the blood-brain barrier. Here is the rational basis for ICIs-based treatment in patients with BM: first, antitumor T cells are activated and home to the brain from extra-cranial sites. Second, tumor neo-vessels are leaky to facilitate ICIs penetrating to stimulate tumor-associated T cells. Prospective trials evaluating ICIs efficacy in previously untreated BM are scarce. It was supported by a few studies of anti-PD-1 mono-therapy that ICIs can induce intra-cranial response.^[16–18]^ Series of retrospective studies provided evidence that intracranial and extra-cranial efficacy of ICIs are comparable.^[16,19]^

Some questions have yet to be resolved regarding how to identify the patients most likely to benefit from ICIs-based therapy, and how to weigh the probability of immunotherapy benefit when concomitant potential positive and negative factors both exist. For this patient, concomitant alterations in immune-related genes were revealed including *CTNNB1* S37F and *ARID2* E1056X. *CTNNB1* S37F is a gain-of-function mutation that could lead to aberrant activation of the WNT/β-catenin signaling, which is enriched in non-T cell inflamed tumors^[20,21]^ and has been linked to lack of benefit of immunotherapy in NSCLC.^[22]^ Whereas, *ARID2* E1056X is a loss-of-function mutation. *ARID2*, encoding a PBAF complex subunit, acts as an immunomodulator. *ARID2* mutation is a potential biomarker positively indicating ICIs effectiveness in melanoma patients.^[23–25]^ Considering that biomarkers are often complex and non-binary, and we only see part of the picture since the potential heterogeneity has not been evaluated, therefore, it is overly simplistic to seek predictive power from a single biomarker. An interconnected network of multiple factors would be the eventual biomarker to form a more complete puzzle.

## 4 Conclusion

In conclusion, our case demonstrated that upfront chemotherapy plus PD-1 inhibitor might be an option for some NSCLC patients of BM harboring *EGFR* exon 20 insertion and high PD-L1 expression/high TMB. Additional insights into gene aberrations provide more information. Further study is needed to validate the predictor involved in responders to ICIs-based therapy with *EGFR* mutations.

## Author contributions

**Conceptualization:** Jingying Nong, Yi Zhang.

**Data curation:** Jingying Nong, Shuyang Yao.

**Resources:** Yanfei Gu, Shuyang Yao.

**Supervision:** Yi Zhang.

**Writing – original draft:** Jingying Nong.

**Writing – review & editing:** Jingying Nong, Yanfei Gu, Yi Zhang.
